# Alveolar adenoma with a single cyst: A case report

**DOI:** 10.3892/mi.2024.140

**Published:** 2024-02-16

**Authors:** Hiroshi Matsui, Takahiro Utsumi, Natsumi Maru, Yohei Taniguchi, Tomohito Saito, Haruaki Hino, Kaori Ishida, Koji Tsuta, Tomohiro Murakawa

**Affiliations:** 1 Department of Thoracic Surgery, Kansai Medical University Hospital, Hirakata, Osaka 573-1010, Japan; 2 Department of Pathology and Laboratory Medicine, Kansai Medical University Hospital, Hirakata, Osaka 573-1010, Japan

**Keywords:** alveolar adenoma, benign lung tumor, tumor with air image, type II pneumocytes, lung cancer

## Abstract

Alveolar adenoma is a rare and benign pulmonary tumor, which originates from type II pneumocytes and is often incidentally identified on radiographic images. Alveolar adenoma presents as a peripleural, solitary and cystic nodule in the lung and may mimic other types of lung tumors, thus rendering its differential diagnosis difficult. Alveolar adenoma is diagnosed based on histopathological and immunohistochemical analyses. The present study describes the case of a 50-year-old male patient with alveolar adenoma. He visited a local doctor ~3 years prior due to left chest pain. A chest computed tomography scan revealed a cystic lesion in segment 8 of the left lung. A nodular shadow appeared in the cyst and gradually increased in size; the patient was thus referred to the authors' hospital. The nodule was well-defined, solitary and solid; thus, lung cancer or aspergilloma were suspected. Thoracoscopic wedge resection was performed as diagnostic therapy. The frozen sections were non-diagnostic, and a pathological examination revealed an alveolar adenoma with no evidence of malignancy and a negative culture. The patient had a good post-operative course, with no sign of recurrence at the follow-up evaluation 46 months later. On the whole, alveolar adenoma is a rare, benign pulmonary tumor that is difficult to diagnose pre-operatively.

## Introduction

Alveolar adenoma (AA) is an exceedingly rare benign lung tumor that was first reported by Yousem *et al* in 1986(1). Patients with AA are usually asymptomatic and the tumor is incidentally detected on chest radiography as a clear solitary pulmonary nodule. When solid pulmonary nodules are >10 mm in size, there is an increased likelihood of surgical intervention due to a higher risk of malignancy and patient anxiety (2,3). A definitive diagnosis of AA depends on histopathology and immunohistochemistry findings as it is difficult to diagnose pre-operatively. Curative treatment consists of surgical resection, with no case of recurrence having been described to date in the literature, at least to the best of our knowledge.

The present study describes the case of a patient with alveolar adenoma and reports on the imaging and pathological differentiation of AA from other lung tumors.

## Case report

**Figure 1:**
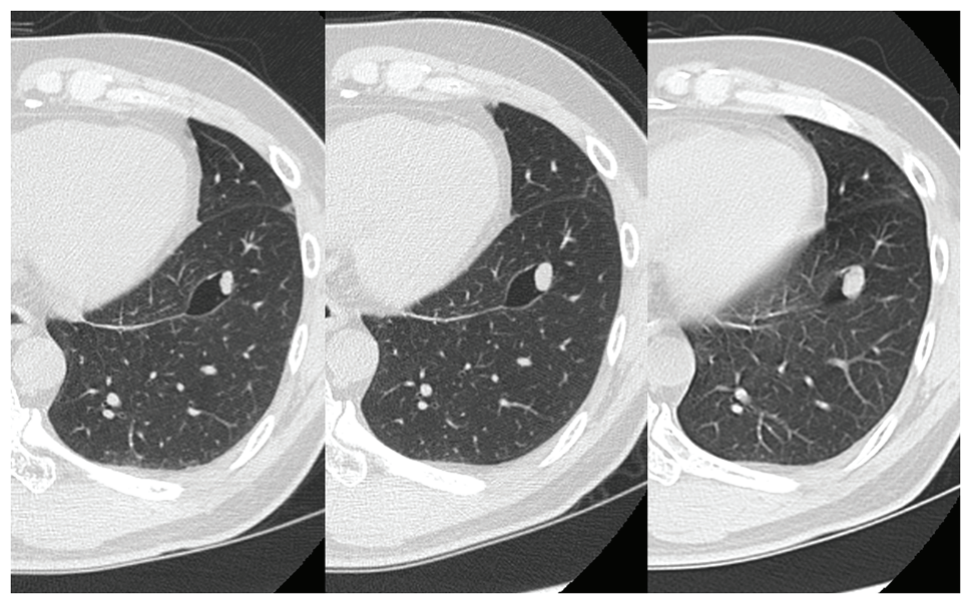
Chest CT illustrating a well-defined nodule in segment 8 of the left lung with a surrounding vacuole, and the nodule increased in size on the CT scan for 2 years (images from left to right indicate the passing of time, respectively).

A 50-year-old male patient presented to his previous doctor with a complaint of chest discomfort. A chest computed tomography (CT) scan revealed a 10-mm solitary nodule with a single cyst in the left lower lobe. The diameter of the pulmonary nodule increased to 14 mm within a span of 2 years (CT scan results shown in Fig. 1). He was referred to Kansai Medical University Hospital (Hirakata, Japan) due to the possibility of lung cancer. His medical history was notable for benign prostatic hyperplasia. A thoracoscopic wedge resection of the left lower lung was performed. The frozen sections were non-diagnostic, and the surgical procedure and postoperative course were uneventful, with no signs of recurrence 4 years post-operatively.

**Figure 2:**
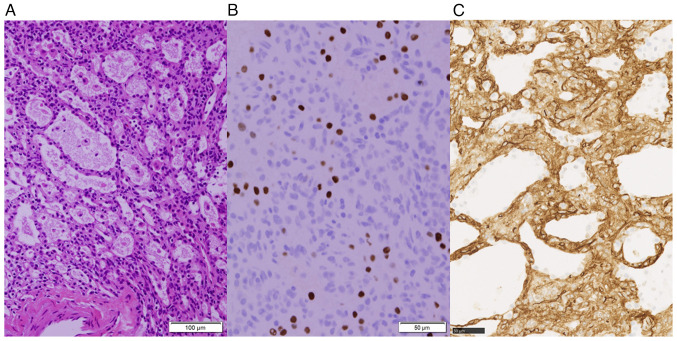
Histopathological findings of the tumor illustrating (A) multiple cystic spaces resembling alveoli (hematoxylin and eosin staining; magnification, x200). (B) Its lumen was covered with hyperplastic type II alveolar epithelial cells, which were thyroid transcription factor-1-positive (immunostaining; magnification, x400). (C) The stroma lacked elastic fibers and contained CD34-positive cells with rounded nuclei and an eosinophilic cytoplasm (immunostaining; magnification, x400).

Overall, the tumor was well-defined and yellowish, measuring 10 mm in a subpleural cyst. Microscopically, the tumor consisted of polycystic structures resembling alveoli filled with pulmonary surfactant. The cyst lining cells were positive for thyroid transcription factor-1 (TTF-1) without atypia, corresponding to type II pneumocytes (Fig. 2). Additionally, the stroma lacked elastic fibers characteristic of alveoli and contained cluster of differentiation 34 (CD34)-positive cells with rounded nuclei and eosinophilic cytoplasm. These cells were negative for TTF-1, CD31, ERG, D2-40, SALL4, BRAF and STAT6. Immunohistochemical analysis was conducted on 4-µm-thick formalin-fixed paraffin-embedded tissue sections (details presented in Table I). Cultures were negative for fungi and tuberculosis, and no malignant cells were noted. Analyses were performed under a light microscope (Olympus Corporation) and whole slide images. Therefore, the final pathological diagnosis was that of AA.

## Discussion

AA is a rare lung tumor with an incidence rate of <1% of all lung tumors, and is classified as an adenoma in the 2015 World Health Organization Classification of lung tumors (4). AA is often asymptomatic and is incidentally detected during imaging examinations, typically exhibiting no tendency to enlarge (5). The majority of patients are middle-aged to elderly, with a slight predominance in the female sex. There is no association between the occurrence of AA and a previous medical history or family history. AA commonly occurs in the middle and lower lobes of the lung and is pathologically characterized by multifocal cystic lesions resembling alveolar cavities, with the lumen lined by TTF-1-positive type II alveolar epithelium (6). In the present study, upon an examination of the patient, it was found that the tumor had similar histopathological features to the cases reported in the literature (6-11).

AAs are characterized by the presence of vacuoles within or around the tumor on imaging (7). It is speculated that alveoli rupture and fuse to create cavities, similar to the cavity formation mechanism observed in lung cancer. This occurs as tumor cells develop toward the bronchiole, forming a unidirectional check-valve system, which results in the accumulation of gas in the alveoli. This phenomenon may explain the cystic lesions observed in the pathology. Therefore, it is important to consider AA as a differential diagnosis in cases of pulmonary nodules with air images. In the case presented herein, the tumor increased in size with the presence of air images, highlighting the necessity to distinguish the tumor from lung cancer or pulmonary aspergilloma.

The diagnosis of AA is challenging when based on small biopsy tissue or frozen sections as it can resemble normal lung parenchyma or mimic malignancy with small glandular spaces lined by regular glandular epithelium (8). Additionally, there are other conditions in the differential diagnosis of AA, including papillary adenoma, sclerosing pneumocytoma and pulmonary hamartoma. Papillary adenoma is characterized by distinctive papillae covered by uniform cuboidal to columnar cells and a heterogeneous epithelial component. The presence of TTF-1 expression in AA can help distinguish it from sclerosing pneumocytoma. Pulmonary hamartoma consists primarily of benign cartilage mixed with a fibrovascular stroma and scattered bronchial glands (9).

The curative treatment for AA is surgical resection, typically performed to rule out malignancy and confirm the diagnosis through postoperative pathology. No recurrence has been reported following complete resection (10). In the case described herein, surgery was concluded with a wedge resection as the tumor was a peripheral lesion that could be completely resected, and the frozen section did not provide a definitive diagnosis. There is a risk of unnecessary extended resection, such as segmentectomy or lobectomy, if the patient is intraoperatively misdiagnosed with lung cancer based in the frozen section; hence, wedge resection is a viable option for small peripheral lesions (11).

In conclusion, AA is a rare, benign lung tumor. When encountering a well-defined solitary nodule with cystic spaces in the peripheral lung, an intraoperative diagnosis can be challenging. Therefore, it is critical to consider the possibility of AA, complete the surgery with a wedge resection and await the final pathological diagnosis.

## Figures and Tables

**Table I:** Antibodies used in the present case report.

Antibody	Clone, cat. no., supplier	Dilution	Incubation	Reaction time	Fully automated IHC system, supplier	Detection system	Antigen retrieval reagent
TTF-1	8G7G3/1, cat. no. IR056, Dako; Agilent Technologies, Inc.	RTU	Low pH: 20 min; room temperature	20 min	Autostainer Link 48, Agilent Technologies, Inc.	Envision FLEX	Envision FLEX Target Retrieval Solution High/Low pH
CD31	JC70A, cat. no. IR610, Dako; Agilent Technologies, Inc.	RTU	High pH: 20 min; room temperature	20 min			
D2-40	D2-40, cat. no. IR072, Dako; Agilent Technologies, Inc.	RTU	High pH: 20 min; room temperature	3 min			
ERG	Cat. no. 418111, Nichirei Biosciences, Inc.	RTU	CC1: 32 min 36˚C	8 min	VENTANA BenchMark ULTRA PLUS, Roche Diagnostics	Optiview DAB universal kit	CC1: VENTANA ULTRA Cell Conditioning Solution
SALL4	6E3, cat. no. H00057167-M03, Abnova	x500	CC1: 32 min 36˚C	16 min			
BRAFV600E	VE1, cat. no. 790-4855, Roche Diagnostics	RTU	CC1: 64 min 36˚C	32 min			
STAT6	YE361, cat. no. ab32520, Abcam	x1,000	CC1: 32 min 36˚C	16 min			

TTF-1, thyroid transcription factor-1; SALL4, Sal-like protein 4; RTU, ready-to-use.

## Data Availability

The datasets used and/or analyzed during the current study are available from the corresponding author on reasonable request.
